# Do Horses Expect Humans to Solve Their Problems?

**DOI:** 10.3389/fpsyg.2012.00306

**Published:** 2012-08-24

**Authors:** C. Lesimple, C. Sankey, M. A. Richard, M. Hausberger

**Affiliations:** ^1^Laboratoire d’Éthologie Animale et Humaine EthoS – UMR CNRS 6552, Station Biologique, Université de Rennes 1Paimpont, France; ^2^Laboratoire d’Éthologie Animale et Humaine EthoS – UMR CNRS 6552, Campus de Beaulieu, Université de Rennes 1Rennes, France

**Keywords:** horses, cognitive skills, human-animal relationship, attention

## Abstract

Domestic animals are highly capable of detecting human cues, while wild relatives tend to perform less well (e.g., responding to pointing gestures). It is suggested that domestication may have led to the development of such cognitive skills. Here, we hypothesized that because domestic animals are so attentive and dependant to humans’ actions for resources, the counter effect may be a decline of self sufficiency, such as individual task solving. Here we show a negative correlation between the performance in a learning task (opening a chest) and the interest shown by horses toward humans, despite high motivation expressed by investigative behaviors directed at the chest. If human-directed attention reflects the development of particular skills in domestic animals, this is to our knowledge the first study highlighting a link between human-directed behaviors and impaired individual solving task skills (ability to solve a task by themselves) in horses.

## Introduction

Domestic animals are highly efficient in detecting human cues, even subtle ones (e.g., Gacsi et al., 2004; Maros et al., 2008; Proops et al., 2009), while close wild relatives tend to perform less well in human related tasks, such as responding to pointing gestures or gaze direction (Hare et al., 2002; Miklosi et al., 2003; Gacsi et al., 2005). It is generally suggested that domestication through genetic and/or experiential processes, may have promoted the development of skills related to the detection of human signals (Hare et al., 1998; Virányi et al., 2008).

Non-domestic captive animals also show some ability to respond to human signals such as pointing gestures (e.g., Miklósi and Soproni, 2006 for a review) or to detect human attentional states (dolphins: Xitco et al., 2004; apes: Hattori et al., 2007; Tempelmann et al., 2011). It is therefore quite possible that captive and domestic animals learn through experience, thanks to interactions with humans during ontogeny and learning/conditioning experiences that include reinforcement of responses to human actions (Udell et al., 2008, 2010, but, see Hare et al., 2010 for comments).

Whether the domestication process or experience with humans (or both) are involved, these processes are generally viewed as a progress in the development of specific sophisticated cognitive skills, such as human signals reading. Indeed, both dogs (Adachi et al., 2007) and horses (Proops et al., 2009; Sankey et al., 2011) have been shown to have expectations of humans’ behavior in a given context. Thus, it was shown that dogs that gaze more at their owner have lower success in problem solving tasks (Topál et al., 1997).

In the present study, we hypothesized that because domestic animals are so attentive to humans and so dependent upon human actions for resources, the counter effect of these abilities may be a lowering of other cognitive skills, such as individual task solving. We investigated whether the performance of domestic horses in an instrumental learning task was influenced by their attention toward the human present.

## Experimental Procedure

### Animals

The experiments were performed between November 2009 and October 2010. The 46 tested horses (24 mares, 22 geldings, 5–23 years old, 8 breeds) were distributed across five French riding schools with similar activities (teaching riders, from beginners to moderate level). Horses were mostly kept singly in 3 m × 3 m straw-bedded individual boxes, fed industrial pellets two or three times a day, and hay once or twice a day. Only seven horses were kept in pasture in group, and fed hay *ad libitum*. All horses had water *ad libitum*.

Horses worked 6–20 h per week in riding lessons involving children and teenagers, with at least one closing day.

### Chest test

**Figure 1:**
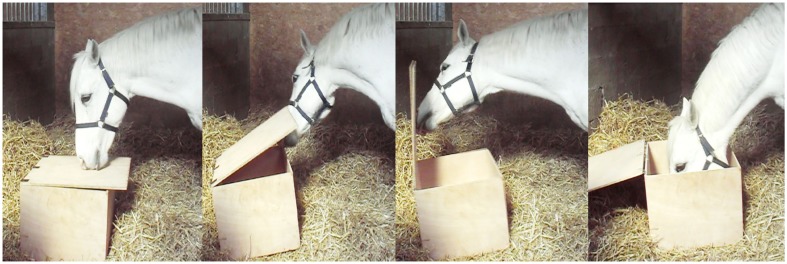
**The chest test: example of behaviors**. Sniffing the lid/Lifting the lid/Opening the chest/Eating food.

The chest test performed here, described in Wolff and Hausberger (1996), is an instrumental learning task, leading to easily measurable performances and presenting correlations with learning abilities at work as assessed by riding teachers (LeScolan et al., 1997). It has been useful to measure cognitive abilities according to different factors such as breed, work (Hausberger et al., 2004), or welfare state (Hausberger et al., 2007). The task consisted of opening a wooden chest (60 cm × 50 cm × 40 cm) in order to find food (Figure 1). To ensure the motivation of the horses for the food, the test was performed in the hour preceding the usual meal time, and the food placed in the chest was their usual food.

In accordance with previous studies, the test was composed of three trials of 3 min, each being preceded by a demonstration. During the demonstrations, the horse was led by the experimenter (C.L. female) with a halter and a rope. After the demonstrations, the experimenter took the rope off, and the trials began. During the trials, the experimenter stood motionless, in a neutral position, with her arms hanging beside the body.

– First trial: the experimenter lifted the lid to open the chest, allowing the horse to see the food inside, and moved the food with the hand.
– Second trial: the experimenter lifted the lid, and then allowed the horse to put its nose inside the chest in order to smell the food.
– Third trial: the experimenter lifted the lid and allowed the horse to introduce its head in the chest in order to eat one mouthful of food.

The total time required to open the chest was recorded (maximum 540 s). The experimenter recorded, using a digital voice recorder (Thomson DK 300).

The following behaviors were recorded:

Exploration behaviors toward the chest, the trough, and/or the experimenter (see Waring, 1983):

– Sniffing: nose near the object of interest, drawing air in through the nostrils.
– Licking: touch an object with the tongue.
– Biting: take the object between the teeth.
– Nibbling: touch an object with lips, moving the lips laterally.
– Pushing: touch the object with the nose or head and pushes on it.

Frustration/excitement behaviors:

– Headshaking: quick vertical movement of the head.
– Startling: quick involuntary movement associated with surprise or alarm.
– Vacuum chewing: chewing with nothing in the mouth (Bergeron et al., 2006).
– Pawing: a foreleg is lifted off the ground, extended in the object direction, and brought back (Waring, 1983).
– Snorting: forceful exhalation through the nostrils (Waring, 1983).
– Whinnying: loud prolonged call associated with alert (Waring, 1983).

Gazes directed to the chest, the trough, and the experimenter were also recorded (i.e., when horse turned its head toward the object of interest and fixed its eyes on it.).

All horses were naïve to this task. Only one experimenter was involved (C. Lesimple), preventing risks of inter observer differences.

### Data and statistical analysis

As data were not normally distributed, we used non-parametric statistical tests (Siegel and Castellan, 1988), with a significance threshold at 0.05.

The homogeneity of the data between the five schools was assessed using the Kruskall–Wallis test (KW). As no differences emerged between the five schools, neither in the time nor in the number of trials required to open the chest (KW, *p* > 0.1 in each case), data from the different schools were pooled.

Fisher’s exact test and Spearman’s correlation tests were used to explore the links between horse performance and behavior during the test. Friedman ANOVAs were used to assess the change during the test (i.e., between the three trials).

## Results

During the hour preceding the meal time, horses were required to open a chest containing their usual food. The test was divided into three trials lasting 3 min each, so that horses had a maximum of 9 min to succeed in opening the chest.

Half of the horses succeeded in opening the chest within three trials (23/46), and most of them showed some interest in the experimenter, from mere gazes (*N* = 38, 82.6%) to exploration (sniffing, nibbling, *N* = 30, 65.2%).

Interest in the experimenter was associated with failure to perform the task, as more than half of the horses that showed exploration behaviors toward the experimenter failed to open the chest (*N* = 18, 60%), while only a third of those that did not (*N* = 5, 31.2%) failed (Fisher’s exact test, *p* = 0.04).

**Figure 2:**
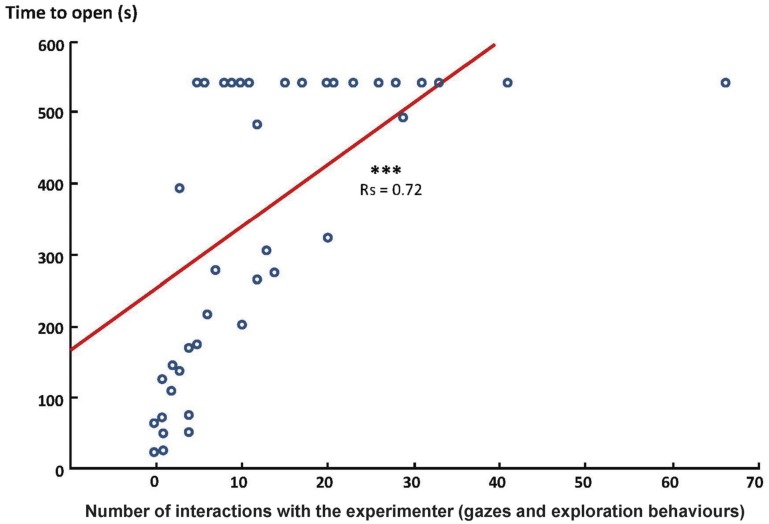
Correlation between the interest of the horse toward the experimenter and the time required to open the chest (Spearman correlation test, ***p < 0.001). Data are represented in number of behaviors and time (s) required to open the chest.

In fact, there was even a clear correlation between horses’ interest toward the experimenter (exploration + gazes) and the time (Spearman’s correlation test: *N* = 46, *r*_s_ = 0.72, *p* = 0.0001; Figure 2) and number of trials (Spearman’s correlation test: *N* = 46, *r*_s_ = 0.71, *p* = 0.0001) required to open the chest. Interestingly, this was not related to a lack of interest in the chest.

Indeed, horses that showed most interest in the experimenter were also those that showed most direct (but inappropriate) investigative behaviors toward the chest (sniffing sides: Spearman correlation test: *N* = 46, *r*_s_ = 0.45, *p* = 0.001, and lid *r*_s_ = 0.35, *p* = 0.02; licking the chest *r*_s_ = 0.6, *p* = 0.003; biting the chest *r*_s_ = 0.31, *p* = 0.03), while being at the same time those that performed the less (in the context) “appropriate” behaviors toward the chest (creeping their nose under the lid, *r*_s_ = −0.37, *p* = 0.001).

Therefore, the “human-directed” horses appeared to investigate more the chest without properly trying to open it, expressing at the same time behaviors that indicated frustration such as head shaking (*r*_s_ = 0.35, *p* = 0.02), snorting (*r*_s_ = 0.34, *p* = 0.02), startling (*r*_s_ = 0.42, *p* = 0.004), and vacuum chewing (*r*_s_ = 0.29, *p* = 0.05).

As the amount of human-directed, trough-directed (Table 1) and frustration behaviors did not change across trials (no differences between trials 1, 2, and 3, *p* > 0.05), it seems that this tendency to depend upon humans and be less efficient in opening the chest could be an intrinsic characteristic of these animals more than a consequence of the waiting for the food.

**Table 1:** **Change of the number of human-directed and trough-directed behaviors across trials for horses that failed to open the chest (*N* = 23)**.

	Human-directed behaviors			Trough-directed behaviors		
	Trial 1	Trial 2	Trial 3	Trial 1	Trial 2	Trial 3
Mean	5.8	7.2	6.4	0.6	0.9	1.1
Standard error	1.4	1.2	1.1	0.3	0.4	0.4
Range of values	(0–32)	(0–27)	(0–20)	(0–2)	(0–9)	(0–8)

## Discussion

Observations of horses faced to a food related instrumental task (opening a chest) showed clear correlations between their interest in the human and success or failure to the task. Interest toward human was associated with increased (“inappropriate”) investigation behaviors toward the chest, frustration behaviors, and failure to open the chest. As it was also correlated with interest toward the usual feeding trough (where humans usually put the food), one interpretation could be that these horses expected humans to pour the food in the feeder as they usually do. They made less “appropriate” real trials to open (creeping their nose under the lid) which may mean that either they did not “understand the task” (i.e., lack of “causal learning”) and relied upon humans to solve the problem and/or they had their attention focused on the humans’ face (monitoring its attentional state, e.g., Sankey et al., 2011) and therefore not on the way the chest could be opened (lifting the lid; i.e., “oversensitivity to human distractor”). In the same way, dogs that were more dependent to their owners (more glancing and following) showed decreased problem solving abilities in a food related task (Topál et al., 1997), suggesting that a strong attachment to humans could lead to an impairment of these abilities.

Horses do not learn to perform a spatial or instrumental task from a human demonstrator (Wolff and Hausberger, 1996) and therefore they had to find the solution to open it by lifting the lid. Half of them succeeded, which is in accordance with some earlier findings in domestic horses (LeScolan et al., 1997; Lesimple et al., 2011). Both genetic and environmental factors (such as type of work) have been shown to influence performances in this task (Hausberger et al., 2004), which means that both the domestication process and experience may be involved in determining the success rate observed. The data here do not allow to determine which kind of experience could be involved. Horses fed with automatic distribution have been shown to exhibit less frustration than those fed manually (Fureix et al., 2011). One could think also that the quality of the human-horse relationship may influence the horses’ interest toward the experimenter (Hausberger et al., 2008; Fureix et al., 2009; Sankey et al., 2010). Only further experiments will bring possible answers. Both may also be involved in horses’ expectations of “human help”. Further studies comparing wild/feral to domestic horses and different feeding conditions in domestic horses should now be performed.

Domestication however cannot fully explain the observed individual differences which may also reflect different levels of dependency on human actions (Topál et al., 1997; Smith and Litchfield, 2010; Jakovcevic et al., 2012). Relation to humans varies individually in horses (e.g., Hausberger et al., 2008), and we suggest that the level of dependency may also vary. What selection and domestication may well have brought is a finer “knowledge” of human behavior but also maybe a decrease of self sufficiency when faced to non-usual challenges.

## Author Contribution Statement

C. Lesimple performed the experiments, analyzed the data and wrote the manuscript. C. Sankey contributed to data analyses and corrected the English. M. A. Richard designed the experiment and contributed to data analyses. M. Hausberger designed the experiments, contributed to data analyses and to manuscript writing. All authors reviewed the manuscript.

## Conflict of Interest Statement

The authors declare that the research was conducted in the absence of any commercial or financial relationships that could be construed as a potential conflict of interest.
